# Anatomy of intergovernmental finance for essential public health services in China

**DOI:** 10.1186/s12889-022-13300-y

**Published:** 2022-05-09

**Authors:** Xiao Tan, Christine Wong

**Affiliations:** 1 Centre for Contemporary Chinese Studies, The University of Melbourne, 761 Swanston Street, Parkville, VIC3010 Australia; 2 East Asian Institute, National University of Singapore, 469A Bukit Timah Road, Tower Block #06-01, Singapore, 259770 Singapore

**Keywords:** Essential public health services, Fiscal equalisation, Intergovernmental finance, China

## Abstract

**Background:**

The Chinese government launched health care reforms in 2009 and introduced a national list of essential public health services (EPHS) as an integral part of the plan to deliver health care for all. EPHS was also built into the national plan to promote the equalisation of public services across the country. A national standard was set for financial input to EPHS. As the services are co-funded by the central and local governments, a robust intergovernmental fiscal system is essential to guarantee that the hundreds of thousands of service providers have adequate financing to meet the service commitment.

**Methods:**

We examined the flow of funds through China’s complex intergovernmental fiscal system to see whether the promise of equal funding for EPHS was implemented, and how the costs were distributed across levels of government. Information was collated from funding documents issued by all levels of governments involved, for a sample that includes the central government, 12 provincial governments, eight prefectural governments and 11 county-level governments. For each level of government, we examined: (i) when and how much funding they disbursed or received from higher levels; (ii) when and how much matching funds were made; and (iii) the allocation rules adopted.

**Results:**

Overall, we found the central government met its commitments for the program on time and in full, and good compliance from local governments in passing through funding from higher levels and as well as meeting their own financial responsibilities. However, we also found the following problems: (i) the involvement of so many levels of government resulted in delays in the disbursement of funds; (ii) the use of outdated population data in calculating required funding resulted in some under-allocation; and (iii) localities that needed funding the most were not well targeted by the distribution of funds.

**Conclusion:**

This study traces how the 2018 subsidy for EPHS was disbursed from the central government to service providers, focusing on the roles played by intermediate levels of subnational governments—provinces, prefectures and counties. In this way, it identifies gaps in the current intergovernmental financing of EPHS and points to areas for further improvement.

**Supplementary Information:**

The online version contains supplementary material available at 10.1186/s12889-022-13300-y.

## Background

The outbreak of the Severe Acute Respiratory Syndrome in 2003 was a wake-up call to the Chinese government. Since then, substantial resources have been poured into the public health system, strengthening public health institutions such as centres for disease control and offering more and better public health services through primary care facilities at the grassroots level [1]. In 2009, with the launch of reforms that aimed to deliver basic health care for all by 2020, the central government introduced a national list of essential public health services (EPHS) [2]. The list covered a wide range of services to be delivered free of charge to users, from traditionally provided essentials such as vaccinations to new services in chronic disease management [3].

Since 2012, the EPHS has also been incorporated into the government’s grand plan to promote equalisation of public services across the country under a new development paradigm that emphasised equalisation and inclusiveness. The goal was to build towards a system where every citizen would have equal access to basic public services [4, 5], starting with the equalisation of funding.

To ensure each citizen receives an equal financial contribution from the government, the central government sets the level of subsidy for EPHS each year. In 2018 it was 55 yuan per capita^1^ [6]. The subsidy for EPHS is co-funded by the central government and different levels of subnational governments. As such, a robust intergovernmental fiscal system (IFS) is essential to guarantee that service providers at the grassroots level, particularly those in poor regions, have access to needed resources. Having recognised this, the Chinese government embarked on a series of reforms to clarify financing responsibilities of central and local governments and updated allocation guidelines to better target allocation of funds to the most needed areas [7–9]. Detailed guidelines have been issued for various types of basic public services, EPHS being one of them [10, 11].

Despite a recognition of the significance of the IFS in the provision of EPHS and more broadly, in supporting the Chinese government’s ambition of equalising public services, to date systematic information on how the system works is scarcely available. Notably, there is limited information on what the roles of intermediate levels of subnational governments—provinces, prefectures and counties—are. This study intends to fill this important gap. By collecting and analysing financing documents from different levels of the Chinese government, this study traces how the 2018 subsidy for EPHS was disbursed from the central government to hundreds of thousands of services providers. The purpose of this study is to evaluate and identify problems in the transmission process.

### The Chinese national package of EPHS

Since its inception, EPHS has grown rapidly. In 2018, the program covered 12 items (Table 1). Some items such as personal health records and health education are targeted at the entire population while other items are targeted at specific groups. For example, vaccinations are mainly for children, and chronic disease management is primarily for hypertensive and diabetic patients. Notably, EPHS does not include medical care. Under EPHS, the management of chronic conditions covers only screening and monitoring (e.g., to monitor blood pressure and blood glucose) [12]. Treatment would come under other programs.

**Table 1:** National list of essential public health services, 2018

Item	Eligible people
1. Personal health records	All permanent residents
2. Health education	All permanent residents
3. Vaccinations	Children aged 0–6 years
4. Child healthcare and management	Children aged 0–6 years
5. Maternity care and management	Pregnant women
6. Aged care and management	People aged 65 years or over
7. Management of chronic conditions	Patients with hypertension and/or diabetes
8. Management of severe mental disorders	Patients with severe mental illness
9. Management of tuberculosis	Patients with tuberculosis
10. Use of traditional Chinese medicines for health maintenance	Children aged 0–3 years and people aged 65 years or over
11. Reporting of and emergency response to infectious diseases	All permanent residents
12. Supporting health inspection activities	All permanent residents

Source: [6, 13]

**Fig. 1:**
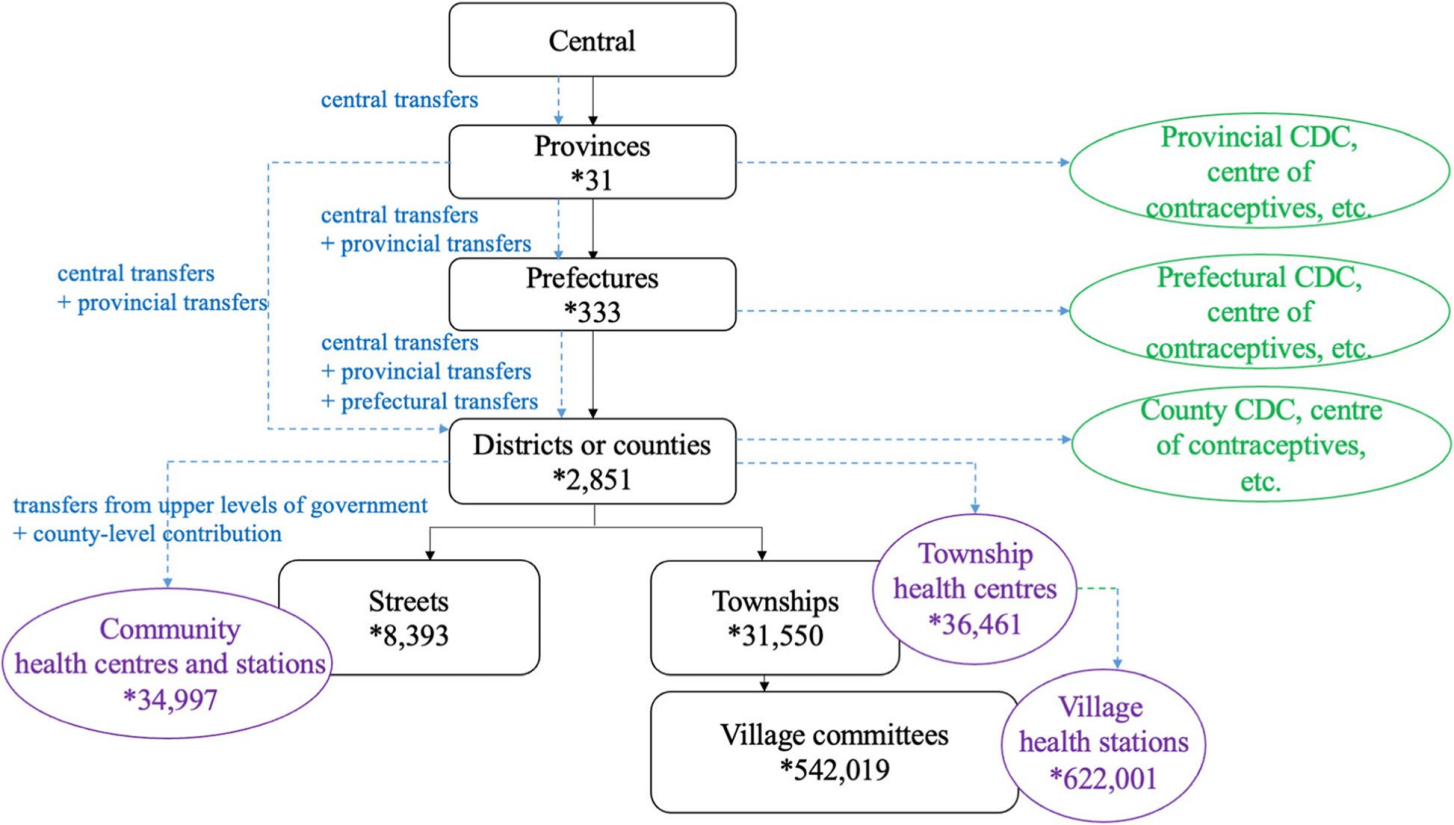
Providing and financing essential public health services. Note: This is a simplified depiction of the administrative structure. ‘Provinces’ represent provinces (22), autonomous regions (5), and provincial level municipalities (4); ‘prefectures’ represent prefectures (7) and prefectural level cities (293), and leagues (3); etc. CDC=Centre for Disease Control. Green circles are public health institutions, and purple circles are primary care facilities. Solid lines represent supervisory relationships and dashed lines represent money flows. Data in this figure are from 2018, collected from [14]

EPHS services are delivered predominantly by primary care facilities, which claim the overwhelming bulk of EPHS funding^2^. These primary care facilities are largely public institutions, organised on a territorial basis (Fig. 1). Rural areas are served by township health centres and village health stations, and urban areas by community health centres and stations (a minority of community health facilities are in rural areas)^3^. The organisation of these primary care facilities is a legacy of the planned economy, guided by the principles of a rational division of responsibility rather than competition, with one township health centre in each township, one village health station in each village, and one community health centre (which may be supported by multiple community health stations) in each neighbourhood (administratively called ‘street’). Each primary care facility is responsible for providing EPHS for people within their catchment area^4^.

The primary care facilities are located at the county level and are the responsibility of county finance. Under China’s IFS, county governments receive transfers from higher levels of government, and these transfers are passed down level-by-level through the administrative hierarchy (Fig. 1)—from the central government to the provinces, from provinces to prefectures or counties and from prefectures to districts or counties; in this transmission process, each level of government also adds their own contribution and has some discretion in making allocation rules. As such, the financing of EPHS involves numerous decision-makers at the subnational level (i.e., provinces, prefectures, and counties). Their decisions all have significant implications, including the financial burden at their lower level(s), to what extent services providers can recover their costs, and whether those most in need receive the resources. This study is a detailed exploration of these issues.

### Problems in the financing of EPHS

While the literature on the financing of EPHS in China is small, it is filled with findings of myriad problems in the program. A few have focused on the inadequacy of government subsidies [1, 15]. Cost analyses of sampled community health centres in Beijing, Shenzhen and Zhuhai consistently showed that the government subsidy was significantly less than the costs of delivering EPHS, and that the low level of subsidy has weakened the incentive for the delivery of EPHS [16–18]. Few studies have focused on intergovernmental financing of EPHS, but some early studies and reports have noted that central funding allocations were not targeted at provinces most in need of financial support [19]. Some found that matching funds from subnational governments were not provided fully or in time [20, 21]. There was a long gap between the time when the funds were allocated by the central government and the time when service providers received the money [22]. In addition, there was a lack of clarity regarding how much different levels of subnational governments should contribute [22, 23]. Contributions from public finance tended to depend on the fiscal capacity of local governments, resulting in unequal patterns [23].

Despite these observations, to our knowledge, none of the previous studies has evaluated intergovernmental financing of EPHS systematically. One of the reasons for this was data limitations since the Chinese government’s effort to disclose more public finance data is a relatively recent development—significant progress was only made after the new budget law in 2014, which requires that all local governments disclose their budgets and final accounts. Benefiting from this progress, we were able to collect fiscal data for EPHS from different levels of government. Furthermore, the earlier observations need to be revised due to new policy developments such as the government’s recent effort to allocate responsibility more precisely to each level of government [10, 11]. Since 2014 reforms have also required early disbursal of transfers at all levels of government [8, 9]. In light of these developments, this study contributes to the literature by providing a detailed picture of how the EPHS subsidy is disbursed from the central government to service providers—identifying gaps and pointing out areas for further improvement.

## Methods

To examine the intergovernmental financing of EPHS, we collected funding documents issued by various levels of government for the year 2018. Our data were significantly shaped by the current progress of data transparency reform. Although all subnational governments disclose their annual budgets and final accounts now, other types of fiscal documents (including funding documents for EPHS) are still not disclosed completely or systematically. However, since EPHS is supported by a specific purpose/earmarked funding, cross uses are not allowed and when reported, EPHS funding is separated from other public health expenditures. This critical setting enables clear and consistent reporting of EPHS funding throughout the transmission process.

Our data collection started with a preliminary round of internet searches using the keywords ‘基本公共卫生服务’ (EPHS), ‘补助资金’ (subsidy), 2018 and three administrative levels— ‘省’ (province), ‘市’ (city) and ‘县’ (county). These Chinese terms were entered into both Baidu and Google search engines to identify units where such information was available^5^. For each locality identified, we attempted to obtain a complete set of funding documents through official government websites. Documents were deemed complete when they covered information on: (i) how much a given locality received from higher levels of government; (ii) how much contribution was made at its own level; and (iii) how much the locality allocated to lower-level units.

Overall, information tended to be closer to complete at the central and provincial levels. At the prefectural level, one practical reason for incomplete information disclosure is the fragmentation of funding. As will be discussed in the next section, central, provincial and prefectural governments all made contributions but tended to split their contributions into different tranches—one for pre-allocation, one for settlement and maybe another one or more for specific purposes. At the prefectural level, there may be five or six different documents, issued at different times, all for the funding of EPHS in 2018. It was common for localities to disclose only the allocation of central and provincial funding while others reported only contributions from their own level^6^. At the county level, it was extremely difficult to access and compile individual funding documents because even more documents were involved after the prefectural level and, perhaps more importantly, data transparency was generally worse at the grassroots level [24, 25]. However, some counties were able to provide summaries of how much they allocated to EPHS in total, including all the funds they received from higher levels of government. The limitation was that such summaries usually did not provide the timing of receipts or disbursements.

Apart from information completeness, another sampling criterion was to seek coverage in all three regions (i.e., eastern, central and western regions) and at different levels of economic development. The search resulted in a final sample of 12, eight and 11 units at the provincial, prefectural and county levels, respectively (Table 2). A full list of the policy documents is provided in Additional File 1.

**Table 2:** Study sample

Province-level units (12)	Qinghai, Sichuan, Henan, Shanxi, Hunan, Hainan, Heilongjiang, Hebei, Fujian, Liaoning, Guangdong and Shanghai
Prefecture-level units (8)	Shangqiu (Henan), Changsha (Hunan), Harbin (Heilongjiang), Yiyang (Hunan), Xi’an (Shaanxi), Hanzhong (Shaanxi), Shantou (Guangdong), Huizhou (Guangdong)
County-level units (11)	Minhang (Shanghai), Zhanyi (Yunnan), Zhaohua (Sichuan), Qidong (Jiangsu), Minquan (Henan), Sui (Hubei), Liuyang (Hunan), Yuhua (Hunan), Luodian (Guizhou), Wugong (Shaanxi) and Chengcheng (Shaanxi)

For each level of government, we examined (i) when and how much funding they received or was allocated from higher levels of government; (ii) when and how much matching contributions were made; and (iii) the allocative rules adopted—how the funding from higher levels of government and their matching funds were allocated across different subordinate units. To facilitate comparison and easier understanding, we report *per capita* government funding. This was derived by dividing original total fiscal data from government sources by the corresponding population in 2018, which was calculated as the average of the population at the end of 2017 and the population at the end of 2018. This may differ from the population data used by the government.

## Results

### From the central government to provinces

In 2018, the central government allocated a total of 41.6 billion yuan for the provision of EPHS (doc-CENT-01, doc-CENT-02). This works out to 30 yuan per capita [22], or 55% of the 55 yuan per capita set as the national standard. The majority of central funding was disbursed in October 2017, with the remaining disbursed in June 2018 (Table 3). This met the Ministry of Finance requirement that the central government should partially pre-allocate earmarked funding two months in advance of the fiscal year covered by the funding (i.e., before 31 October) [8]. In calculating the funding requirements for 2018, the central government used the year-end 2016 population data (doc-CENT-02). This resulted in the actual per capita central allocation falling slightly below target in most provinces (due to population growth; Table 3).

**Table 3:** Per capita central government allocation of funding, 2018

Province	Per capita GDP/national average	Planned per capita total central funding (yuan), stated in policy documents	Actual per capita central funding (yuan)		
			Total	Allocated in October 2017	Allocated in June 2018
**Total**	**1**	**N/A**	**29.8**	**24.2**	**5.6**
Beijing (East)	2.2	5.5	5.5	4.5	1.0
Shanghai (East)	2.1	5.5	5.5	4.5	1.0
Tianjin (East)	1.9	5.5	5.5	4.5	1.0
Jiangsu (East)	1.8	11	11.0	9.0	2.0
Zhejiang (East)	1.5	5.5	5.4	4.4	1.0
Fujian (East)	1.4	27.5	27.2	22.0	5.2
Guangdong (East)	1.3	9.5	9.3	7.4	1.9
Shandong (East)	1.2	22	21.9	17.7	4.2
Inner Mongolia (West)	1.1	44	43.9	35.8	8.2
Hubei (Central)	1.0	33 or 44	36.0	29.4	6.6
Chongqing (West)	1.0	44	43.5	35.1	8.3
Shaanxi (West)	1.0	44	43.6	35.4	8.2
Liaoning (East)	0.9	16.5	16.4	13.6	2.8
Jilin (Central)	0.9	33 or 44	34.2	28.2	6.0
Ningxia (West)	0.8	44	43.4	35.1	8.4
Hunan (Central)	0.8	33 or 44	36.7	29.8	6.9
Hainan (Central)	0.8	33	32.6	26.5	6.2
Henan (Central)	0.8	33 or 44	36.9	30.2	6.7
Xinjiang (West)	0.8	44	42.8	30.4	12.4
Sichuan (West)	0.8	44	43.7	35.5	8.2
Hebei (Central)	0.7	33	32.7	26.6	6.1
Anhui (Central)	0.7	33 or 44	36.9	29.9	7.0
Qinghai (West)	0.7	44	43.5	35.3	8.2
Jiangxi (Central)	0.7	33 or 44	37.1	30.8	6.3
Shanxi (Central)	0.7	33 or 44	36.4	29.5	6.8
Tibet (West)	0.7	44	42.8	34.3	8.5
Heilongjiang (Central)	0.7	33	32.9	27.2	5.7
Guangxi (West)	0.6	44	43.4	35.2	8.2
Guizhou (West)	0.6	44	43.5	35.4	8.1
Yunnan (West)	0.6	44	43.6	35.4	8.2
Gansu (West)	0.5	44	43.7	35.6	8.1

Data source: Fiscal data were collected from doc-CENT-01 and doc-CENT-02 (Additional File 1). Population and per capita GDP data were collected from [14]

The allocation of central funding is by province and follows the redistributive principle employed for all central government transfers that targets more to poor areas. The redistribution is coarse, though. This framework employs a tripartite division of the 31 provinces into eastern, central and western regions, each with almost 500 million population. Under this division, central government transfers are tilted towards the western provinces, which, on average but not uniformly, are the poorest (Table 3). The central provinces are also recipients of substantial transfers, whereas the eastern provinces receive far less. In 2018, the central government set a target of providing 44 yuan per capita to western provinces, 33 yuan per capita to central provinces and between 5.5 and 27.5 yuan per capita to eastern provinces (doc-CENT-02)^7^.

The adoption of the region-based approach means that the allocation was not very precisely linked to economic resources. For example, the 12 western provinces that received the highest level of central subsidies were not necessarily the least economically developed (Table 3). Such an approach disadvantaged some central and eastern provinces with less developed economies. For example, Heilongjiang (classified as a central province) and Liaoning (classified as an eastern province) obtained significantly less than the provinces with a similar level of economic development (Table 3). This means that sub-provincial governments in these two provinces were under greater pressure to meet the spending requirement given their more limited resources. The finding echoes those of earlier studies, which suggest that Shandong obtained less subsidy compared to western provinces with similar levels of economic development and is disadvantaged by its location in the eastern region [19, 26].

**Fig. 2:**
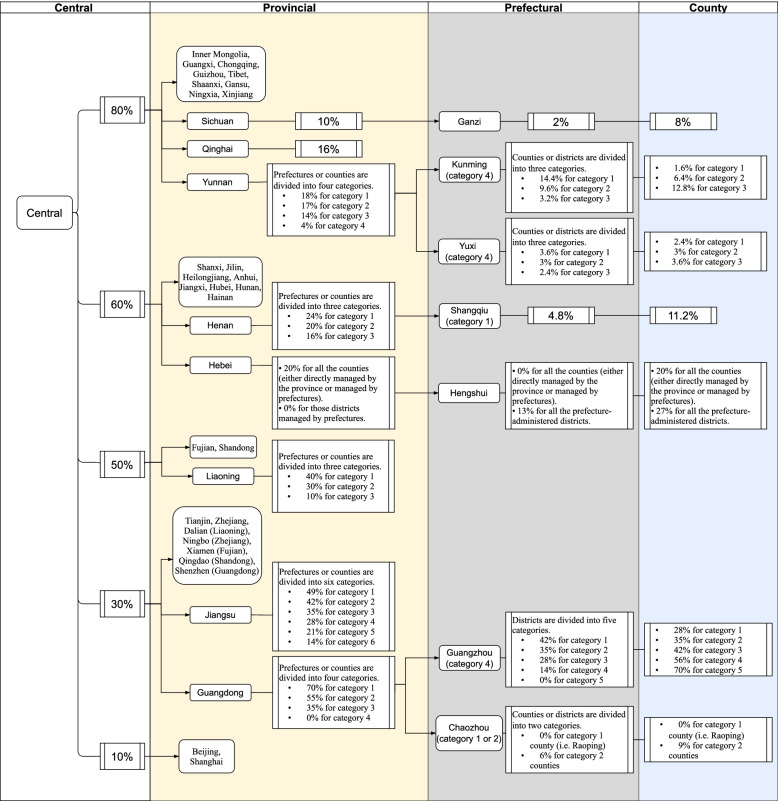
Government guidelines for subsidy allocation, effective from 1 January 2019. Note: Categories are ranked from poorest to richest, with category 1 denoting the poorest group. Data source: Central rules are from [11]. Rules adopted by subnational governments are compiled and calculated by the authors; the data sources are omitted here but can be provided upon request

In the central government’s newly introduced guideline in 2019, provinces are now divided into five categories, with the central government sharing 80%, 60%, 50%, 30% and 10% of the total government subsidy (see Fig. 2). However, a comparison between the new guideline and the 2018 allocation plan (see the third column of Table 3) shows great similarity.

### From provinces to prefectures or counties

**Fig. 3:**
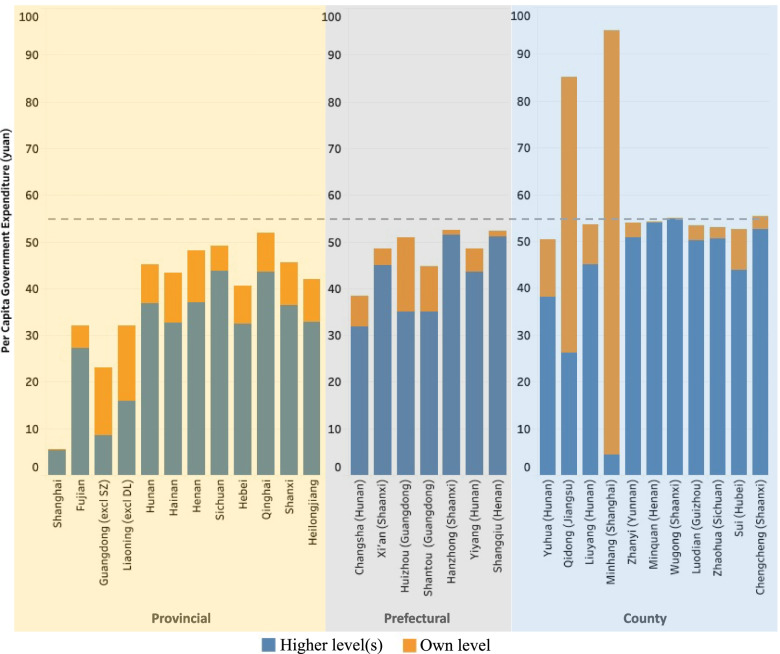
Per capita government contributions in sample provinces, prefectures, and counties, 2018. Note: For Harbin (Heilongjiang), the statistical yearbook only reports the hukou population (which differs from the permanent population used for other units). Therefore, per capita fiscal data cannot be derived, and the prefecture is omitted here. Data source: Fiscal data were collected from government documents listed in Additional File 1. Population and per capita GDP data were collected from [14], supplemented with a mix of provincial and prefectural statistical yearbooks and local statistical bulletins

**Fig. 4:**
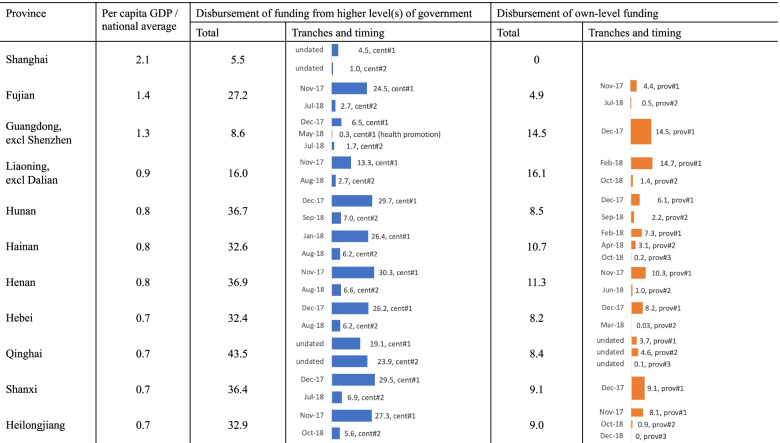
Government allocation of funding in sample provinces, 2018. Note: †Sichuan does not report information on when funding was received or disbursed. The province is thus omitted here. ‡In Fujian and Qinghai, central funding was combined with provincial funding and not separately reported. Per capita central funding in Table 3 was used to derive per capita provincial funding. §Per capita GDP data for Guangdong and Liaoning are for the whole provinces, not excluding Shenzhen and Dalian. For the third tranche of provincial funding in Heilongjiang, no additional funding was given, but final adjustments were made (i.e., deducting funding from some sub-provincial units to reward others). Data source: Fiscal data were collected from the provincial-level documents listed in Additional File 1 (i.e., doc-PROV-01 to doc-PROV-36). Data for the population in Shenzhen and Dalian were collected from http://data.stats.gov. Other population data and per capita GDP data were collected from [14]

Provincial-level governments distribute funding both from the central funding received and from own resources to the prefectures or counties under their jurisdiction. Among the ten provinces that specified the amount of central funding they received (Fujian and Qinghai combine the central and provincial funding and report them together), all of them passed through the exact amount that the central government claimed was distributed (Fig. 3 and Table 3). They did not keep the money for other purposes. In terms of timing, the provincial governments sent through the two tranches of central funding in the periods of November 2017‒January 2018 and July‒October 2018, respectively (Fig. 4), at around one to four months after funding was reportedly disbursed by the central government.

Provincial governments may also make additional contributions—in one to three tranches—that were sometimes combined with central funding. Following the process adopted by the central government, provincial governments also pre-allocated most of their funding between November 2017 and February 2018, which was timely for use in 2018. The only exception was Qinghai, where less than half of the central and provincial funding was disbursed in the first tranche. Since there was no clear date on the relevant policy documents published on the provincial government websites, it was difficult to determine when the two tranches of central and provincial funding were disbursed and whether the second tranche was too late for use at the grassroots level.

The amount of provincial addition to the central funding varied across provinces (see Figs. 3 and 4). Among the 12 provinces in our sample, the Liaoning provincial government added the most to the central funding—around 16 yuan per capita. This was followed by Fujian, which contributed approximately 5 yuan per capita. At the other extreme, the Shanghai provincial/municipal government made no financial contribution, passing the entire funding responsibility to the county (district) level.

When calculating the amount to be allocated to lower levels, provincial governments also used population data from an earlier year. Among those provinces reporting the year of population data used, Shanxi, Hebei and Hainan used data from 2016, while Hunan used 2017 data. In these four provinces, the actual per capita funding was around 1% lower than the planned per capita funding calculated using the average population data in 2018.

While some provinces specified rules only for prefectures (e.g., Qinghai), others went further by specifying how much each *county* should receive (e.g., Shanxi, Hebei, Hainan, Hunan and Fujian). Allocation rules varied but they can be divided into two categories.

- (i) Equal treatment: In Heilongjiang, Hebei and Qinghai, each prefecture or county was given the same level of central and provincial funding per capita. For example, in Heilongjiang, each prefecture was given 33 yuan in central subsidy and 9 yuan in provincial subsidy per capita. This is irrespective of their differences in economic resources, where per capita GDP in 2018 was 4.7 times in the richest prefecture compared to the poorest.
- (ii) Differentiated treatment: Other provinces adopted a differentiated approach in allocating funds that mimics the central government’s. In Shanxi, for example, the poor counties were given 35.3 yuan per capita from the central subsidy while all other counties were given just 26.5 yuan per capita. In Hainan, the more developed cities (i.e., Haikou, Sanya and Yangpu) were given a smaller provincial subsidy (8.8 yuan per capita) compared to other areas (13.2 yuan per capita). In contrast to the equal approach, the differentiated approach takes economic development into consideration and tilts more funding support to those areas that have less economic resources.

Following the central government’s lead, provincial governments have tried to clarify responsibilities at the provincial and lower levels further. Despite this, the complex structure and diverse arrangements were largely retained (Fig. 2). Some provinces dealt only with prefectures and left further allocation arrangements to prefectures (e.g., Liaoning and Qinghai), while others set allocation rules for some or all counties directly. Some provinces adopted a flat structure (e.g., Heilongjiang, Qinghai), setting the same per capita funding for all prefectures or counties, while others tilted allocations to poorer localities (e.g., Guangdong and Liaoning). Overall, rather than radically changing existing arrangements, the new rules appeared to be incremental in nature with only minor changes.

### From prefectures to counties

The role played by prefectures in managing EPHS funding varied significantly across provinces since some provincial governments set rules for all counties. In these cases, prefectures were either bypassed when it came to decision-making or are simply expected to implement provincial policies. Other provincial governments only dealt with prefectures and not local governments lower in the hierarchy, leaving space for prefectures to determine how to allocate central and provincial funding. Some provinces preferred adopting a middle ground—with the provincial governments setting allocation rules for some counties (such as fiscally and directly managed counties) while leaving prefectures with authority to determine allocation for prefecture-administered districts.

Cross-checking was done for all the prefectures that separately report the amount of central funding received (Harbin, Changsha and Yiyang report some or all their central and provincial funding), and the numbers were consistent with those reported in the central documents. Data from Shangqiu, Shantou and Huizhou were cross-checked, since their provincial documents (i.e., from Henan and Guangdong) were also collected, and the prefectural figures are found to be consistent with those reported in provincial documents. Based on the evidence available in this study, the prefectural governments have complied well with provincial policies, distributing the exact amount of central and provincial funding to lower levels.

**Fig. 5:**
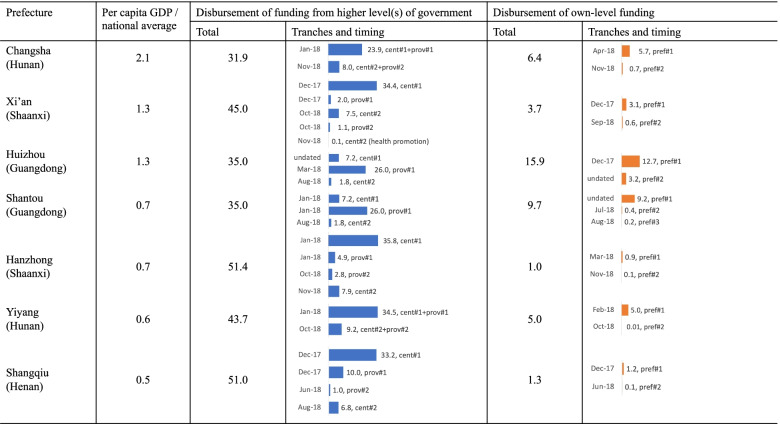
Government allocation of funding in sample prefectures, 2018. Note: †For Harbin (Heilongjiang), the statistical yearbook only reports the hukou population (which differs from the permanent population used for other units). Therefore, per capita fiscal data cannot be derived and the prefecture is omitted here. ‡Per capita GDP data are for entire prefectures, not excluding the units as suggested in the first column. §In Changsha and Yiyang, central funding was combined with provincial funding and not separately reported. For Changsha, the central and provincial funding covers areas excluding Ningxiang and Liuyang while the prefectural funding covers all areas in Changsha. Therefore, different population data were used to derive per capita fiscal data. Data source: Fiscal data were collected from the prefectural-level documents listed in Additional File 1 (i.e., doc-PREF-01 to doc-PREF-34). Other data were collected from a mix of provincial and prefectural statistical yearbooks, supplemented with county-level statistical bulletins

In addition to disbursing the funds from higher levels, prefectural governments also made their own contributions, at differing amounts (Fig. 5)^8^. As Fig. 3 illustrates, there was no clear association between per capita GDP and prefectural contribution. Instead, prefectures in those provinces receiving a relatively low level of central subsidy needed to fill the gap themselves. The governments in Huizhou and Shantou contributed more, despite having a poorer economy compared to Xi’an.

In terms of timing, most prefectural governments disbursed the first tranche of central, provincial and prefectural funding in the period of December 2017‒April 2018 (Fig. 5). As for the second tranche of funding, some prefectural governments (e.g., Harbin and Changsha) made the funds available to county-level governments only around the end of 2018.

Population data are still essential for prefectural governments in determining how much they allocate to districts or counties. Following the practice of central and provincial governments, prefectural governments also tended to use population data from an earlier year. Hanzhong, Shangqiu, Xi’an, Shantou and Huizhou used population data at the end of 2016; Changsha and Yiyang used population data at the end of 2017; and Harbin reported that they used the ‘confirmed population in 2018’, without specifying where the population data came from and how it was confirmed (doc-PREF-14).

Among prefectures in the sample, Xi’an was affected the most by population growth. In the funding documents, the Xi’an government used the population figure taken at the end of 2016, which was 7.32 million. The population in 2018 (calculated as the average of the population at the end of 2017 and that at the end of 2018) was 7.68 million, which was 5% higher than the figure used by the government. According to relevant policies, Xi’an was given 44 yuan per capita from the central government and another 3.3 yuan per capita from the provincial government (doc-PREF-18). However, due to the population growth, the actual per capita funding was only 41.9 and 3.1 yuan per capita, respectively, around 4‒5% below the target level.

The guideline for subsidy allocation also varied across prefectures. Mirroring the patterns at the provincial level, they can be divided into two broad groups—equal treatment or differentiated treatment. Prefectural governments may either follow their provinces’ approaches or not. For example, Harbin followed the equal approach used by Heilongjiang.

In contrast, while the Guangdong province adopted the differentiated approach, Huizhou applied a flat structure to allocate prefectural funding. Following the practice of central and provincial governments, prefectural governments have made an effort to clarify responsibilities at the prefectural and county levels further (Fig. 2). Again, these newly introduced rules were mostly similar to existing rules in 2018. As the last column shows, after the layers of central, provincial and prefectural allocations, the financial burden on county governments varies significantly. A relatively poor locality could have a relatively heavy financial burden (e.g., the poorest counties in Shangqiu are paying 11.2%, higher than many counties in richer prefectures).

### At the county level

At the county level, some counties received funds directly from their provincial government while others received funds from their prefectural government. The per capita central funding in Zhaohua (Yunnan), Minquan (Henan), Wugong and Chengcheng (both in Shaanxi) were 42.7, 43.2, 44.0 and 44.2 yuan, close to or equal to the 44 yuan per capita central subsidy the three provinces should have received in 2018 (see Table 3). This shows that central funding successfully reached the county level. Data from Minquan (Shangqiu, Henan), Liuyang (Changsha, Hunan) and Yuhua (Changsha, Hunan) were cross-checked, since their prefectural and provincial documents were also collected, and consistency in numbers was found across all levels.

**Fig. 6:**
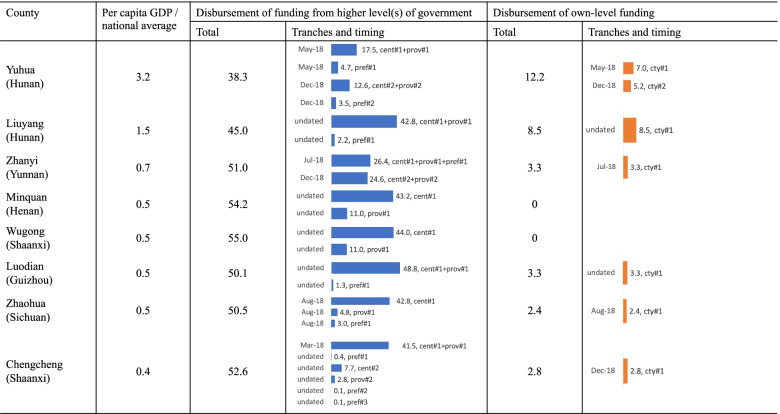
Government allocation of funding in sample counties, 2018. Note: Minhang (Shanghai), Sui (Hubei), Qidong (Jiangsu) and Fufeng (Shaanxi) do not report information on when funding was received or disbursed. These counties are thus omitted here. Data source: Fiscal data were collected from the county-level documents listed in Additional File 1 (i.e., doc-CTY-01 to doc-CTY-13). Population data for Liuyang were collected from Hunan statistical yearbooks. Other population data and per capita GDP data were collected from local statistical bulletins

**Fig. 7:**
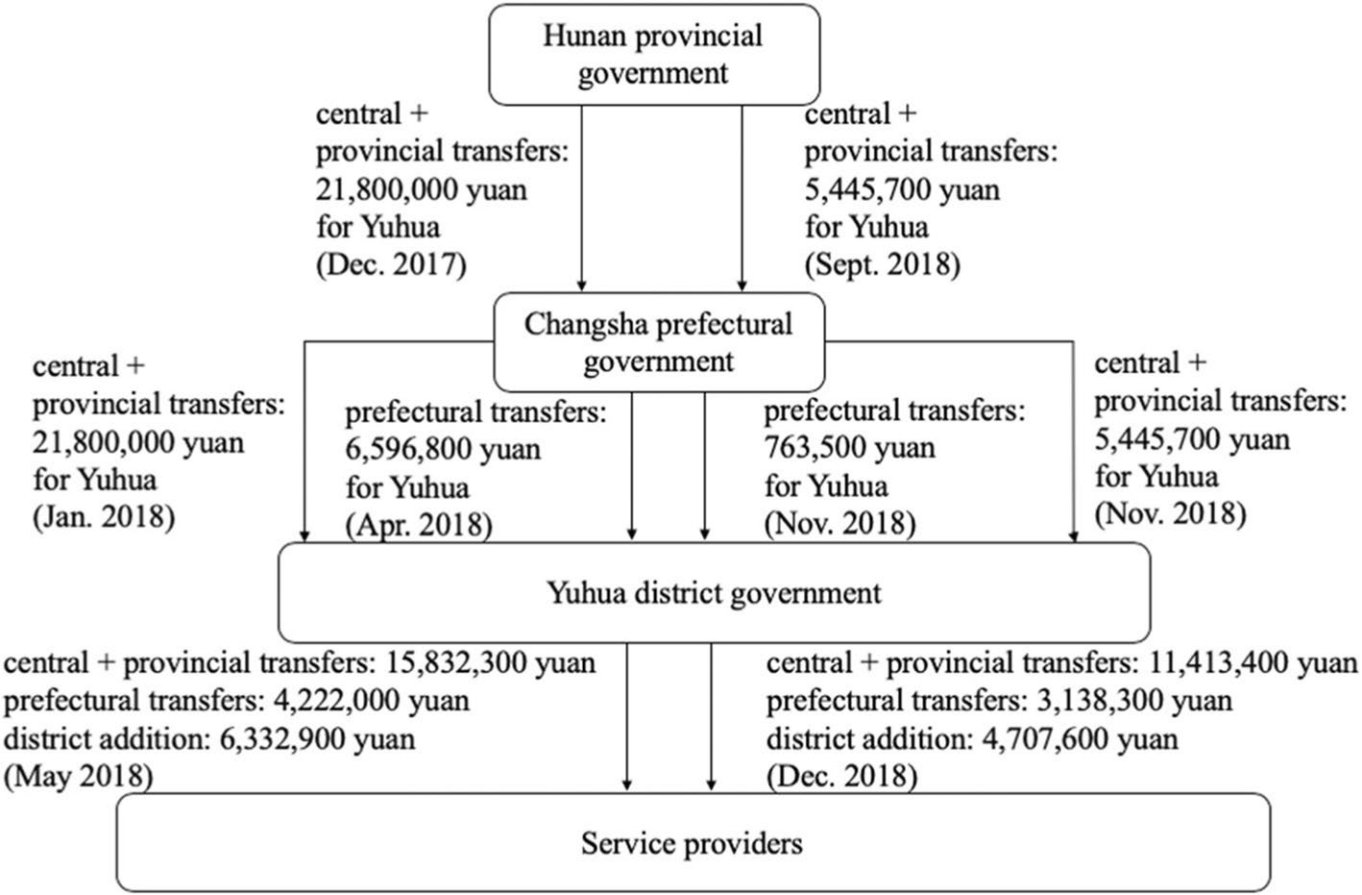
Transmission of funds (Yuhua District, Hunan). Data source: doc-PROV-25, doc-PROV-26, doc-PREF-04, doc-PREF-05, doc-PREF-06, doc-PREF-07, doc-PREF-08, doc-CTY-09, doc-CTY-10

Although all the allocated funding was eventually disbursed, there were some issues with timing. Not all counties reported the time of fund disbursement, but among those that did (Fig. 6), the first tranche of central funding was disbursed between March and August 2018—around five to 10 months after the funding was first allocated by the central government (i.e., October 2017). Some counties chose to allocate only part of the funds received from higher levels of government first, resulting in an even longer delay. In Yuhua’s case, the Hunan government allocated 21.8 million yuan of central and provincial funding for the Yuhua District in December 2017 (see Fig. 7). The exact amount was distributed by the Changsha government to the Yuhua District in January 2018. However, the Yuhua government only disbursed 15.83 million in May 2018 to service providers. Similarly, while the Changsha government allocated 6.6 million yuan of prefectural funding in April 2018, the Yuhua government only disbursed 4.2 million yuan in May 2018. The remainder of the first tranche of central, provincial and prefectural funding was allocated in December 2018, together with the second tranche of funding. Therefore, although the Yuhua government eventually transmitted all the central, provincial and prefectural funding, there were some severe delays in the funding received by service providers.

The amount of county-level governments’ contribution varied significantly across localities (Fig. 3). The county-level governments in Minquan and Wugong made no additional financial contributions because their higher level governments had already contributed around 55 yuan per capita. The problem identified was that funding from higher levels of government was not very precisely targeted to those areas that needed it the most. Although the per capita GDP in Sui (Hubei) was similar to that in Zhaohua (Sichuan) and Chengcheng (Shaanxi), the county government in Sui had to contribute significantly more than the other two counties (see Fig. 6).

The total subsidy in most counties reviewed was close to the required level of 55 yuan per capita (Fig. 3). Qidong and Minhang were two exceptions where the district government contributed significantly more and drove the actual per capita total spending to higher levels. Receiving less than 55 yuan per capita funding can be expected for two reasons. First, the government tended to use population data in 2016 or 2017. The population growth during these one or two years lowered the actual per capita spending. Second, the provincial and prefectural governments set aside some money for province or prefectural health promotions and family planning purposes. Yuhua had the lowest per capita spending, at 50.3 yuan per capita, which was about 9% lower than the required level. A closer look into the funding documents suggests that this was mainly due to the population data used. Local statistical bulletins suggest that there were 907,700 permanent residents in 2018. However, the local government used the number of 836,400 to represent the population, without explaining how they obtained this figure. One possibility was that the government used the average of permanent residents and *hukou* (or ‘household individual’, a Chinese system of household registration) population (which was around 720,000 in 2018). Such an approach resulted in a large number of non-*hukou* permanent residents not being taken into account for government funding.

## Discussion

Under China’s IFS, the financing of public services is highly fragmented and administratively complicated; the EPHS is no exception. The funds are passed down hierarchically, either in the central‒provincial‒prefectural‒county order or in the central‒provincial‒county order. Each subnational level of government allocates the funds received from higher level governments and makes additional contributions. The funding is fragmented further by the practice of disbursement in multiple tranches.

As the findings illustrate, this complex and fragmented financing pattern has resulted in unpredictable financial burden and inequality at the county level—the final destination for the overwhelming bulk of EPHS funding. The allocation of central transfers is primarily based on macro regions and insufficiently fine-tuned to target all poor provinces. Then the vast diversity in the rules for subsidy allocation across provinces and prefectures lead to the poorer localities not necessarily receiving more transfers from higher levels of government. In this way, although both the central and many subnational governments have adopted a differentiated approach in tilting more resources to poorer areas, the final outcome does not entirely reflect the equalisation principle. The finding echoes those of earlier studies [26, 27], which collectively suggest that the complex structure of the Chinese intergovernmental fiscal system impedes resources flows. A substantial reform of the IFS would be essential to support the equalisation of public services.

Perhaps more importantly, until now, the government’s allocation rules have only aimed to achieve equalisation in financial *input*. Such a focus is unlikely to produce equal outcomes. On the demand side, the current allocation rules have not taken into account the differences in health status across regions and their differing needs for EPHS. The disease burden is likely to vary significantly across localities, making the delivery of services more challenging for some. However, same targets are universally applied to different localities.

On the supply side, it is actually unclear how much is being spent on EPHS. As a legacy from the planned economy era, on top of EPHS funding, primary care facilities still receive line item budget from the government, primarily used to cover part of the salaries for their staff. The government is also financially responsible for subsidising operational deficits and purchasing infrastructure and needed equipment for publicly owned primary care facilities [28]. In return, primary care facilities implement government mandates—including delivering medical services at discounted rates and EPHS services free of charge. Collectively, these settings enable the government to impose a uniform per capita government subsidy for EPHS services without nuanced considerations of needs and cost of services. However, the mixed use of financial arrangements also makes the real cost obscure because other types of government subsidies received by primary care facilities can be used to cross-subsidies the delivery of EPHS activities in forms such as labour cost and capital depreciation.

Further, the real cost of EPHS is likely to differ significantly across localities given that it can be expensive to hire people to provide services in large cities where living cost is high. But those urban providers with large population sizes may benefit from economies of scale. In the current system, the only major flexibility to accommodate these variations is that those localities in those economically developed areas are expected to draw from their local resources to provide more funding. However, this leaves space for local discrepancies and does not guarantee sufficiency [16–18].

Given that China is a vast country with significant regional differences with a highly complex IFS, there are important final notes to add. As mentioned previously, given the data limitations, we relied on a sampling approach. At higher levels, data are relatively comprehensive and should offer good representability. At lower levels, however, although we purposedly sampled localities from different regions and of different economic development, the sample size is small considering the large number of sub-provincial governments in China. Therefore, the findings for lower-level governments are more tentative and will require further data to test. We note that the Chinese government has been making continuous efforts in data transparency reform. So far, impressive progress has been made in the disclosure of budgets and final accounts (less so for sub-items such as EPHS funding discussed in this study). We believe that more systematic research can be done following our approach as the reform progress and in this way, our study sets an important starting point for further investigation.

Our paper has focused on one specific aspect of China’s EPHS program—why and how the fragmented and complex nature of China’s public finance system imposes barriers for a program designed to bring equality in EPHS for the population. There are other critical dimensions, such as the actual cost of EPHS services, the efficiency of EPHS spending across localities and providers, and ultimately how EPHS contributes to improvements in population health outcomes. Knowledge of these issues will contribute to a more comprehensive evaluation of China’s EPHS program. Also, given China’s EPHS is relatively new, we consider a complementary and fruitful direction to conduct substantial comparisons between China’s EPHS and the corresponding public health services in other countries. The comparisons would make the Chinese case more relevant to other countries, showing a different approach in funding and delivering public health services and the associated benefits and costs.

## Conclusions

Overall, we found good compliance in local governments’ transmission of funds from higher levels of government and in fulfilment of their own financial responsibilities. At the grassroots level, the total government subsidy received was either higher or close to the national requirement. However, this finding needs to be interpreted with caution, as the study sample covers only those local governments that disclosed their funding information publicly. It is possible that localities that do not have good compliance may also tend to avoid disclosing information.

Despite the data limitations, we identified three important issues in the government’s disbursement process. First, there were significant and multiple delays in the time it took for funds to be disbursed from one level to the next. The central government allocated the first tranche of funding in October 2017. This was transmitted to the next level by provincial governments between November 2017 and January 2018; by prefectural governments between December 2017 and February 2018; and finally by county governments between March and August 2018—totalling around five to ten months after the funds were first allocated by the central government. Such a long lag has financial implications that affect service providers’ incentives and behaviour.

Second, the central and local governments used outdated population data (from 2016 or 2017) to calculate the EPHS funds needed for 2018. This resulted in a gap between the actual and targeted per capita spending. At higher levels, the gap was relatively small because population growth within one or two years was limited. However, the effect was experienced unequally in different localities. Large cities were likely to experience faster population growth, so when accurate and updated population data were not used, it left a significant number of people out of the equation. In particular, migrant workers were most likely to be left out due to their high mobility. In our sample, Xi’an city (Shaanxi) and the Yuhua District (Changsha, Hunan) were affected the most by population issues. Both are in metropolitan areas where population growth is fast, and the proportion of migrant workers is high. In Xi’an’s case, the government’s use of 2016 data resulted in the actual per capita funding received for EPHS falling around 5% lower than the target level. In Yuhua’s case, the local government used population data that was averaged from the number of permanent residents and the *hukou* population, resulting in the actual per capita spending falling around 10% lower than the required level.

Finally, the system is placing unequal financial burden across localities. The current government financing structure, combined with different allocation rules adopted by different provincial and prefectural governments, had complicated financial implications. At the central-provincial level, the allocation rule is roughly equalising across huge regions. After the additional layers of differing provincial and prefectural allocations, the distributional outcomes are even more complicated and unpredictable. As such, a local government may either benefit or be disadvantaged by allocation rules made by higher levels of government and a relatively poor locality could have a relatively heavy financial burden.

In 2018, the government introduced reforms to allocate responsibility more precisely to each level of government. These reform efforts resulted in more systematic documentation of guidelines for subsidy allocations for EPHS in publicly available documents. From this perspective, the government has made good progress in promoting more transparency and clarity. However, these reforms can be considered very incremental in nature. Compared to the practice in 2018 (as documented in this study), the new reforms (effective from 1 January 2019) did not introduce any radical changes and thus have not addressed the problems identified in this study.

## Supplementary Information

**Additional file 1.** List of policy documents reviewed.

## Data Availability

All data generated or analysed in this study are included in this paper or Additional File 1.

## Abbreviations

CDC: Centre for Disease Control
EPHS: Essential public health services
IFS: Intergovernmental fiscal system

## Footnotes

1 Localities with more resources may contribute more to top-up the national levels.

2 Although public health institutions are also involved in some EPHS activities (such as health education), their role is relatively very small when compared to primary care facilities. Thus, they claim an almost ignorable portion of EPHS funding (note: these public health institutions provide other types of public health services and receive funding from other government programs). To illustrate, in Sichuan province in 2018, the central government transferred 43.7-yuan-per-capita EPHS funding to the provincial level, of which around 0.4% was spent by various provincial public health institutions, and all the remaining part was disbursed to lower levels of government.

3 Health centres are significantly larger in scale (in terms of space, equipment, and health professionals) when compared with health stations.

4 The two-tiered organisational structure also means that health centres and stations have overlapping catchment areas (e.g., one township is usually covered by one township health centre and several village health stations) and, thus, they share the EPHS workload.

5 Although Google and Baidu search engines were used, all documents were obtained directly from the government’s official website. The search engines were used mainly for efficiency reasons (because at the sub-provincial levels, most governments do not disclose such information at all).

6 In some localities, EPHS are categorised under poverty alleviation. This is not completely correct because EPHS target the entire population instead of only the poor. The ‘mis-categorisation’ is perhaps motivated by local governments’ intention to exaggerate their spending on poverty alleviation, a reform area highly prioritised by the central government. Due to the policy prioritising poverty alleviation, the funding documents for EPHS tend to be more complete in these places.

7 Some designated areas in the central provinces also receive 44 yuan per capita in central subsidies.

8 As a side note, the areas covered by prefectural contributions may differ from the areas covered by central and provincial governments. For example, the Changsha government was responsible for distributing central and provincial funding to their area excluding Ningxiang and Liuyang. These two county-level cities received central and provincial funding from the Hunan provincial government instead. However, when making prefectural funding, the Changsha government included these two cities.
