# Supplementary material for: Anatomy of intergovernmental finance for essential public health services in China
Source: BMC Public Health. 2022 May 9;22:914. doi: 10.1186/s12889-022-13300-y (PMC9080974; doi:10.1186/s12889-022-13300-y)
Supplement: Supplementary file 1 — Additional file 1. List of policy documents reviewed. [file 12889_2022_13300_MOESM1_ESM.docx]

Additional file 1 List of policy documents reviewed

| Code | Name |
| --- | --- |
| *Central level* | |
| doc-CENT-01 | 关于提前下达2018年公共卫生服务补助资金预算指标的通知 [*Notice to allocate the budget quota for the 2018 subsidy for public health services in advance*] |
| doc-CENT-02 | 关于下达2018年公共卫生服务补助资金（基本公共卫生服务）预算的通知 [*Notice to allocate the 2018 budget subsidy for public health services (essential public health services)*] |
| *Provincial level* | |
| 1. Guangdong | |
| doc-PROV-01 | 关于提前下达2018年计划生育等5项中央财政补助资金的通知 [*Notice to allocate five central financial subsidies including the family planning subsidy in 2018 in advance*] |
| doc-PROV-02 | 关于提前下达2018年省级财政卫生计生转移支付资金的通知 [*Notice to allocate the 2018 provincial transfer payment funds for public health services and family planning in advance*] |
| doc-PROV-03 | 关于安排2018年中央补助公共卫生服务等项目资金的通知 [*Notice to arrange the funds for public health services and other projects subsidized by the central government in 2018*] |
| doc-PROV-04 | 关于安排2018年第二批中央财政补助基本公共卫生服务项目资金的通知 [*Notice to arrange the second batch of funds for essential public health services projects subsidized by the central government in 2018*] |
| 1. Liaoning | |
| doc-PROV-05 | 关于提前下达2018年中央财政公共卫生服务补助资金的通知 [*Notice to allocate the 2018 central government subsidy for public health services in advance*] |
| doc-PROV-06 | 关于批复2018年本级年初预算基本公共卫生服务补助资金的通知 [*Notice to approve the subsidy for essential public health services in the budget at the provincial level at the beginning of 2018*] |
| doc-PROV-07 | 关于下达2018年中央财政基本公共卫生服务补助资金的通知 [*Notice to allocate the 2018 central government subsidy for essential public health services*] |
| doc-PROV-08 | 关于下达2018年省财政基本公共卫生服务补助资金的通知 [*Notice to allocate the 2018 provincial subsidy for essential public health services*] |
| 1. Shanghai | |
| doc-PROV-09 | （中央）公共卫生服务补助资金（基本公共卫生服务项目）[*(Central government) subsidy for public health services (essential public health services projects)*] |
| doc-PROV-10 | 2018年财政决算 [*Final accounts for the budget in 2018*] |
| 1. Shanxi | |
| doc-PROV-11 | 关于提前下达中央2018年基本公共卫生服务补助资金预算指标的通知 [*Notice to allocate the budget quota for the 2018 central government subsidy for essential public health services in advance*] |
| doc-PROV-12 | 关于提前下达省级2018年基本公共卫生服务补助资金预算指标的通知 [*Notice to allocate the budget quota for the 2018 provincial subsidy for essential public health services in advance*] |
| doc-PROV-13 | 关于下达2018年中央基本公共卫生服务补助资金的通知 [*Notice to allocate the 2018 central government subsidy for essential public health services*] |
| doc-PROV-14 | 2018年财政决算 [*Final accounts for the budget in 2018*] |
| 1. Sichuan | |
| doc-PROV-15 | 关于2018年基本公共卫生服务财政补助资金安排情况的公告 [*Notice on the arrangement of the 2018 subsidy for essential public health services*] |
| 1. Hebei | |
| doc-PROV-16 | 关于提前下达中央2018年基本公共卫生服务补助资金预算指标的通知 [*Notice to allocate the budget quota for the 2018 central government subsidy for essential public health services in advance*] |
| doc-PROV-17 | 关于提前下达2018年省级公共卫生服务补助资金（第一批）预算指标的通知 [*Notice to allocate the budget quota for the 2018 provincial subsidy for public health services (first batch) in advance*] |
| doc-PROV-18 | 关于下达2018年省级公共卫生服务补助资金（第二批）的通知 [*Notice to allocate the 2018 provincial subsidy for public health services (second batch)*] |
| doc-PROV-19 | 关于下达2018年中央公共卫生服务补助（基本公共卫生服务）结算资金的通知 [*Notice to allocate the settlement funds for the 2018 central government subsidy for public health services* (essential public health services)] |
| 1. Hainan | |
| doc-PROV-20 | 关于下达2018年中央财政基本公共卫生服务项目补助资金（第一批）的通知 [*Notice to allocate the 2018 central government subsidy for essential public health services (first batch)*] |
| doc-PROV-21 | 关于下达2018年省级财政基本公共卫生服务项目补助资金（第一批）的通知 [*Notice to allocate the 2018 provincial subsidy for essential public health services (first batch)*] |
| doc-PROV-22 | 关于下达2018年中央财政基本公共卫生服务项目补助资金（第二批）的通知 [*Notice to allocate the 2018 central government subsidy for essential public health services (second batch)*] |
| doc-PROV-23 | 关于下达2018年省级财政基本公共卫生服务项目补助资金（第二批）的通知[*Notice to allocate the 2018 provincial subsidy for essential public health services (second batch)*] |
| doc-PROV-24 | 关于下达2018年省级财政基本公共卫生服务项目补助资金（第三批）的通知[*Notice to allocate the 2018 provincial subsidy for essential public health services (third batch)*] |
| 1. Hunan | |
| doc-PROV-25 | 关于提前下达2018年基本公共卫生服务补助资金预算指标的通知 [*Notice to allocate the budget quota for the 2018 subsidy for essential public health services in advance*] |
| doc-PROV-26 | 关于下达2018年基本公共卫生服务补助资金的通知 [*Notice to allocate the 2018 subsidy for essential public health services*] |
| 1. Heilongjiang | |
| doc-PROV-27 | 关于提前告知2018年中央和省级财政基本公共卫生补助资金预算指标的通知 [*Notice to announce the budget quota for the 2018 central and provincial subsidy for essential public health services in advance*] |
| doc-PROV-28 | 关于下达2018 年中央和省级财政基本公共卫生服务补助资金(第二批)的通知 [*Notice to allocate the 2018 central and provincial subsidy for essential public health services (second batch)*] |
| doc-PROV-29 | 关于下达2018年基本公共卫生中央和省级财政补助清算资金的通知 [*Notice to allocate the settlement funds for the 2018 central and provincial subsidy for essential public health services*] |
| 1. Henan | |
| doc-PROV-30 | 关于提前下达2018年公共卫生服务补助资金预算指标的通知 [*Notice to allocate the budget quota for the 2018 subsidy for public health services in advance*] |
| doc-PROV-31 | 关于下达2018年基本公共卫生服务中央补助资金的通知 [*Notice to allocate the 2018 central government subsidy for essential public health services*] |
| doc-PROV-32 | 关于下达2018年省级基本公共卫生服务补助资金（第二批）的通知 [*Notice to allocate the 2018 provincial subsidy for essential public health services (second batch)*] |
| 1. Fujian | |
| doc-PROV-33 | 关于提前下达2018年基本公共卫生服务和基层医疗卫生机构运转省级补助资金的通知 [*Notice to allocate the 2018 provincial subsidy for essential public health services and the operation of healthcare organisations at the grassroots level in advance*] |
| doc-PROV-34 | 关于下达2018年基本公共卫生等项目省级补助资金的通知 [*Notice to allocate the 2018 provincial subsidy for essential public health services and other projects*] |
| 1. Qinghai | |
| doc-PROV-35 | 关于提前下达2018年医疗卫生补助资金预算指标的通知 [*Notice to allocate the budget quota for the 2018 subsidy for healthcare and public health services in advance*] |
| doc-PROV-36 | 关于下达2018年度基本公共卫生服务补助资金（第二批）的通知 [*Notice to allocate the 2018 subsidy for essential public health services (second batch)*] |
| *Prefectural level* | |
| 1. Shangqiu, Henan | |
| doc-PREF-01 | 关于提前下达2018年基本公共卫生服务补助资金的通知 [*Notice to allocate the 2018 subsidy for essential public health services in advance*] |
| doc-PREF-02 | 关于下达2018年基本公共卫生服务补助资金（第二批）的通知 [*Notice to allocate the 2018 subsidy for essential public health services (second batch)*] |
| doc-PREF-03 | 关于下达2018年基本公共服务中央补助资金的通知 [*Notice to allocate the 2018 central government subsidy for essential public services*] |
| 1. Changsha, Hunan | |
| doc-PREF-04 | 关于拨付2018年中央和省补助基本公共卫生服务项目资金的通知 [*Notice to allocate the 2018 central and provincial subsidy for essential public health services projects*] |
| doc-PREF-05 | 关于拨付2018年中央和省财政补助基本公共卫生服务项目资金的通知 [*Notice to allocate the 2018 central and provincial fiscal subsidy for essential public health services projects*] |
| doc-PREF-06 | 关于拨付2018年市级基本公共卫生服务第一批补助资金的通知 [*Notice to allocate the first batch of the 2018 prefectural subsidy for essential public health services*] |
| doc-PREF-07 | 关于拨付2018年市级基本公共卫生服务第二批补助资金的通知 [*Notice to allocate the second batch of the 2018 prefectural subsidy for essential public health services*] |
| doc-PREF-08 | 关于拨付2018年市级基本公共卫生服务专项补助（乡村医生岗位补贴）的通知 [*Notice to allocate the 2018 prefectural special subsidy for essential public health services (salary subsidy for rural doctors)*] |
| 1. Yiyang, Hunan | |
| doc-PREF-09 | 关于下达2018年基本公共卫生服务中央 省财政补助资金的通知 [*Notice to allocate the 2018 central and provincial subsidy for essential public health services*] |
| doc-PREF-10 | 关于下达2018年基本公共卫生服务补助资金的通知[*Notice to allocate the 2018 subsidy for essential public health services*] |
| doc-PREF-11 | 关于下达第一批基本公共卫生服务和计划生育事业费市级补助资金的通知[*Notice to allocate the first batch of prefectural subsidy for essential public health services and family planning*] |
| doc-PREF-12 | 关于下达2018年基本公共卫生服务第二批市级补助资金的通知[*Notice to allocate the second batch of the 2018 prefectural subsidy for essential public health services*] |
| 1. Harbin, Heilongjiang | |
| doc-PREF-13 | 关于提前告知2018年中央和省级财政基本公共卫生补助资金的通知 [*Notice to announce the 2018 central and provincial subsidy for essential public health services in advance*] |
| doc-PREF-14 | 关于下达2018年中央和省级财政基本公共卫生服务补助资金（第二批）的通知 [*Notice to allocate the 2018 central and provincial subsidy for essential public health services (second batch)*] |
| doc-PREF-15 | 关于预拨2018年基本公共卫生服务市级财政补助资金的通知 [*Notice to allocate the 2018 prefectural subsidy for essential public health services in advance*] |
| doc-PREF-16 | 关于下达2018年基本公共卫生服务项目市级财政结算补助资金的通知 [*Notice to allocate the settlement funds for the 2018 prefectural subsidy for essential public health services*] |
| 1. Xi’an, Shaanxi | |
| doc-PREF-17 | 关于2018年提前预下转移支付的通知 [*Notice to allocate the 2018 transfer payments in advance*] |
| doc-PREF-18 | 关于结算下达2018年基本公共卫生服务项目中省补助资金的函 [*Letter on the settlement for the allocation of the 2018 provincial subsidy for essential public health services*] |
| doc-PREF-19 | 关于下达2018年中央公共卫生（健康素养促进）补助资金的通知 [*Notice to allocate the 2018 central government subsidy for public health services (promoting health literacy)*] |
| doc-PREF-20 | 关于下达2018年基本公共卫生服务项目补助资金（第二批）的函 [*Letter on the allocation of the 2018 subsidy for essential public health services (second batch)*] |
| 1. Hanzhong, Shaanxi | |
| doc-PREF-21 | 关于下达2018年基本公共卫生服务中省补助资金的通知 [*Notice to allocate the 2018 provincial subsidy for essential public health services*] |
| doc-PREF-22 | 关于下达2018年基本公共卫生服务补助资金的通知 [*Notice to allocate the 2018 subsidy for essential public health services*] |
| doc-PREF-23 | 关于下达2018年中央基本公共卫生服务（健康素养促进）补助资金的通知 [*Notice to allocate the 2018 central government subsidy for essential public health services (promoting health literacy)*] |
| doc-PREF-24 | 关于下达2018年省级基本公共卫生服务补助资金的通知 [*Notice to allocate the 2018 provincial subsidy for essential public health services*] |
| doc-PREF-25 | 关于下达2018年基本公共卫生服务市级补助资金的通知 [*Notice to allocate the 2018 prefectural subsidy for essential public health services*] |
| 1. Shantou, Guangdong | |
| doc-PREF-26 | 关于提前下达2018年计划生育等5项中央财政补助资金的通知 [*Notice to allocate the 2018 central government subsidies for five projects including family planning in advance*] |
| doc-PREF-27 | 关于提前下达2018年省级财政卫生计生转移支付资金的通知 [*Notice to allocate the 2018 provincial transfer payments for public health services and family planning in advance*] |
| doc-PREF-28 | 关于下达2018年第二批中央财政补助基本公共卫生服务项目资金的通知 [*Notice to allocate the 2018 central government subsidy for essential public health services (second batch)*] |
| doc-PREF-29 | 关于提前下达2018年基本公共卫生服务项目市级财政补助经费的通知 [*Notice to allocate the 2018 prefectural fiscal subsidy for essential public health services in advance*] |
| doc-PREF-30 | 关于下达2018年度全市健康素养促进行动项目经费的通知 [*Notice to allocate the 2018 prefectural funds for promoting health literacy*] |
| doc-PREF-31 | 关于下达2018年基本公共卫生服务项目第三批市级补助资金的通知 [*Notice to allocate the third batch of the 2018 prefectural subsidy for essential public health services*] |
| 1. Huizhou, Guangdong | |
| doc-PREF-32 | 关于下达2018年省级财政卫生计生转移支付资金的通知 [*Notice to allocate the 2018 provincial transfer payments for public health services and family planning*] |
| doc-PREF-33 | 关于下达2018年第二批中央财政补助基本公共卫生服务项目资金的通知 [*Notice to allocate the second batch of the 2018 central government subsidy for essential public health services*] |
| doc-PREF-34 | 关于提前下达2018年基本公共卫生服务项目市财政补助资金的通知 [*Notice to allocate the 2018 prefectural subsidy for essential public health services in advance*] |
| *County level* | |
| 1. Minhang district, Shanghai | |
| doc-CTY-01 | 2018年公共卫生服务经费分配的说明 [*Explanations for the allocation of public health services funding in 2018*] |
| 1. Zhanyi district, Qujing city, Yunnan | |
| doc-CTY-02 | 关于下达2018年基本公共卫生服务项目中央和省级补助资金预算指标的通知 [*Notice to allocate the budget quota for the 2018 central and provincial subsidy for essential public health services*] |
| doc-CTY-03 | 关于下达2018年中央和省市区级基本公共卫生服务项目经费指标的通知 [*Notice to allocate the quota for the 2018 central, provincial, prefectural and district-level project funds for essential public health services*] |
| 1. Zhaohua district, Guangyuan, Sichuan | |
| doc-CTY-04 | 2018年基本公共卫生服务等3个项目资金使用方案 [*Fund utilization plan for three projects including essential public health services in 2018*] |
| 1. Qidong city, Nantong city, Jiangsu | |
| doc-CTY-05 | 2018年基本公共卫生服务经费管理和使用情况 [*Management and utilization of funds for essential public health services in 2018*] |
| 1. Minquan county, Shangqiu city, Henan | |
| doc-CTY-06 | 2018年基本公共卫生服务补助资金分配情况公告公示 [*Announcement of distribution of the 2018 subsidy for essential public health services*] |
| 1. Sui county, Sui prefecture, Hubei | |
| doc-CTY-07 | 2018年度基本公共卫生服务预拨资金明细 [*Details of the 2018 funds allocated in advance for essential public health services*] |
| 1. Liuyang city, Changsha city, Hunan | |
| doc-CTY-08 | 2018年度基本公共卫生服务项目资金绩效评价 [*The 2018 performance evaluation of funds for essential public health services projects*] |
| 1. Yuhua district, Changsha city, Hunan | |
| doc-CTY-09 | 关于预拨2018年中央、省、市和区级基本公共卫生服务补助资金的通知 [*Notice to allocate the 2018 central, provincial, prefectural and district-level subsidy for essential public health services in advance*] |
| doc-CTY-10 | 关于拨付2018年中央、省、市和区财政基本公共卫生服务补助资金的通知 [*Notice to allocate the 2018 central, provincial, prefectural and district-level fiscal subsidy for essential public health services*] |
| 1. Luodian county, Qinnan prefecture, Guizhou | |
| doc-CTY-11 | 关于拨付2018年基本公共卫生服务项目结算补助经费的通知 [*Notice to allocate the 2018 subsidy for the settlement of projects for essential public health services*] |
| 1. Wugong county, Xianyang city, Shaanxi | |
| doc-CTY-12 | 2018年基本公共卫生服务项目资金分配方案 [*Allocation plan for the 2018 funds for essential public health services projects*] |
| 1. Chengcheng county, Weinan city, Shaanxi | |
| doc-CTY-13 | 关于下达2018年基本公共卫生服务补助资金的通知 [*Notice to allocate the 2018 subsidy for essential public health services*] |
